# Reverse‑sequence endoscopic nipple‑sparing mastectomy with direct‑to‑implant breast reconstruction and air inflation adjustment technique in patients with large or severely ptotic breast: a single-center prospective cohort study

**DOI:** 10.1097/JS9.0000000000002389

**Published:** 2025-04-10

**Authors:** Hui Dai, Xiaoman Cao, Hao Wu, Faqing Liang, Yanyan Xie, Kawun Chung, Qing Zhang, Tianyuan Li, Zhenggui Du

**Affiliations:** a Department of General Surgery, West China Hospital, Sichuan University, Chengdu, China; b Breast Center, West China Hospital, Sichuan University, Chengdu, China

**Keywords:** breast cancer, cohort study, direct-to-implant breast reconstruction, large or ptotic breast, reverse-sequence endoscopic nipple-sparing mastectomy

## Abstract

**Background:**

The applicability of reverse-sequence endoscopic nipple-sparing mastectomy (R-E-NSM) with direct-to-implant breast reconstruction (DIBR) is worth exploring in patients with large or severely ptotic breasts (LSPB) who were not recommended or even considered contraindicated in open NSM, conventional endoscopic NSM, and robotic NSM. The study aimed to compare the safety and aesthetic outcomes between patients with LSPB and non-LSPB (NLSPB) undergoing R-E-NSM with DIBR.

**Materials and methods:**

The single-center prospective cohort study enrolled 562 patients undergoing R-E-NSM and DIBR. Surgical safety, aesthetic outcomes, and oncologic safety were compared between patients with LSPB and NLSPB.

**Results:**

After propensity score matching, 88 LSPB patients and 256 NLSPB patients were included (median [interquartile range] follow-up time: 21.0 [13.8, 32.4] vs. 23.0 [10.6, 32.2] months, *P* = 0.889). The mastectomy weight was significantly higher in the LSPB group (576.6 ± 144.8 g vs. 330.1 ± 105.7 g, *P* < 0.001). There were no significant differences in any complications (27.3% vs. 22.7%, *P* = 0.381), major complications (6.8% vs. 3.1%, *P* = 0.230), minor complications (20.5% vs. 21.1%, *P* = 0.889) and implant-related complications (21.6% vs. 24.6%, *P* = 0.566) between the LSPB and the NLSPB groups. In the LSPB group, the Ueda scores in patients with air inflation adjustment technique (AIAT) were better than those without AIAT, though no significant difference (*P* = 0.110). Compared to the NLSPB group without AIAT, the LSPB group with AIAT had similar Ueda scores (*P* = 0.870) and a significantly higher increase in BREAST-Q scores of breast satisfaction (*P* = 0.004). Oncologic outcomes had no significant difference between the two groups (all *P* > 0.05).

**Conclusion:**

R-E-NSM with DIBR and AIAT provides comparable surgical safety and aesthetic outcomes for patients with LSPB and NLSPB, offering a new option for LSPB patients.

## Introduction

As breast cancer treatment concepts evolve and aesthetic demands rise, nipple-sparing mastectomy (NSM) is gaining popularity with broadening indications^[^1,2^]^. However, patients with large or severely ptotic breasts (LSPB) are prone to surgical complications after NSM, such as skin flap and nipple-areola complex (NAC) necrosis/ischemia, wound dehiscence, and surgical site infections due to vascular injury, heavy implant weights, prolonged surgery times and so on^[^3,4^]^. Besides, aesthetic outcomes are not consistently satisfactory for unpredictable asymmetry, malposition, and conspicuous scars^[^5,6^]^. Therefore, the prevailing opinion discourages NSM with direct-to-implant breast reconstruction (DIBR) in LSPB patients or even views it as a contraindication^[^7,8^]^. Caution is especially warranted for those with a suprasternal notch-to-nipple distance (SND) exceeding 25 cm^[^9^]^. For the patients, the NSM is commonly combined with the wise-pattern skin-reducing technique (WST), which reportedly escalates surgical risks, injuries, and costs^[^10,11^]^.

Due to the protective effect of the incision-free breast surface on the skin flap and NAC blood supply and the promotion of breast aesthetics, endoscopic NSM (E-NSM) theoretically helps to address the above problems in patients with LSPB^[^12–14^]^. However, the practical application of E-NSM faces many problems, such as the high technical difficulty, long learning curve, and long operation time caused by the difficulty in operating space establishment and instrument interference^[^15–17^]^. Many researchers have turned to robotic NSM (R-NSM), which has a clear and realistic three-dimensional imaging system and flexible robotic instruments. The technical difficulty is relatively reduced, but there are still challenges, such as prolonged operative time, increased cost, and availability of the robotic surgical platform^[^8,18,19^].^ More critically, the commonly reported breast reconstruction (BR) techniques in conventional endoscopic surgery and conventional robotic surgery are nearly total subpectoral^[^19–23^]^. A consensus of leading expert surgeons states that robotics are only indicated for patients with small to moderate-sized, non to mildly ptotic breasts, while ptotic breasts or cup size D and above are considered relatively contraindicated^[^8^]^. Due to the lack of evidence of safety and effectiveness, the FDA has not approved robotic-assisted devices for breast cancer surgery and advises caution in their use for mastectomy^[^24^]^. Therefore, the application of E-NSM and R-NSM in patients with LSPB is facing a dilemma.

Following extensive research and clinical practice, our team has developed an innovative reverse-sequence endoscopic NSM (R-E-NSM) with DIBR^[^25–27^]^. This pioneering technique leverages the expansive force of gas to form a universal retractor and employs an innovative reverse dissection sequence from deep to superficial layers, effectively overcoming the traditional challenges that have impeded the advancement of endoscopic and robotic BR (Supplemental Digital Content 1, available at: http://links.lww.com/JS9/E56, Supplemental Digital Content Video 1, available at: http://links.lww.com/JS9/E56; Supplemental Digital Content 2, available at: http://links.lww.com/JS9/E57, Supplemental Digital Content Video 2, available at: http://links.lww.com/JS9/E57). It significantly enhances surgical efficiency, potentially enabling 24-hour discharge^[^25,27,28^]^. With the “parachute” method for TiLoop® Bra suture and placement, dual-plane BR and prepectoral BR can be easily accomplished, besides total subpectoral BR, as diverse as open surgery^[^26,29,30^]^. Combined with the original air inflation adjustment technique (AIAT), the wrinkled skin and muscle on the surface of the implant are flattened, returning the breast and NAC to their natural status, and then aesthetic outcomes can be further optimized. Consequently, in the context of the above novel techniques, LSPB may no longer be a contraindication of implant-based BR (IBBR). This study aims to prospectively analyze the safety and aesthetic outcomes of the R-E-NSM with DIBR and AIAT in patients with LSPB to explore the potential for expanding the indications for NSM with DIBR. This prospective cohort study has been reported in line with the STROCSS guidelines^[^31^]^, Supplemental Digital Content 3, available at: http://links.lww.com/JS9/E58.

## Methods

### Ethical review

The study was approved by the Biomedical Ethics Committee of West China Hospital (approval number: 2021-592) on 23 July 2021. All patients signed informed consent forms and agreed to have their photos or videos published.

**HIGHLIGHTS**

- In this study, patients with large or severely ptotic breasts (LSPB) undergoing reverse-sequence endoscopic nipple-sparing mastectomy (R-E-NSM) with direct-to-implant breast reconstruction (DIBR) were compared with those without LSPB to explore the surgical safety, aesthetic outcomes, and oncologic safety of R-E-NSM with DIBR in LSPB patients.
- There were no significant differences in surgical complications, implant-related complications, and oncologic outcomes between the LSPB and the NLSPB groups undergoing R-E-NSM with DIBR.
- Integration of air inflation adjustment technique (AIAT) can significantly improve breast satisfaction (BREAST-Q scores) in LSPB patients, achieving aesthetic outcomes comparable to NLSPB groups (Ueda scores).
- R-E-NSM with DIBR and AIAT can bring the same surgical safety and aesthetic outcomes for patients with LSPB compared to patients with NLSPB, which offers a new or superior option for patients with LSPB and challenges contraindications for conventional endoscopic and robotic techniques.

### Study design and patients

**Figure 1.**
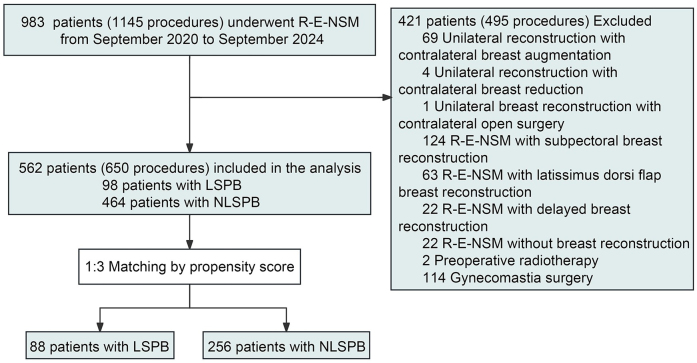
The flowchart of participant selection. DIBR, direct-to-implant breast reconstruction; LSPB, large or severely ptotic breasts; NLSPB, non-large or severely ptotic breasts; PSM, propensity score matching; R-E-NSM, reverse-sequence endoscopic nipple-sparing mastectomy.

The data for this study were derived from a single-center prospective cohort study registered at www.chictr.org.cn (no. ChiCTR2100047081) about comparing breast surgery in day and inpatient units. A total of 983 patients (1145 procedures) underwent R-E-NSM from September 2020 to September 2024 at West China Hospital, a tertiary A teaching general hospital in China. According to the following criteria, 562 patients (650 procedures) undergoing R-E-NSM with DIBR were enrolled. The inclusion criteria: (i) tumors no larger than 5 cm, classified as cN0-2, with no clinical or radiographic signs of skin or chest wall infiltration and distant metastasis; or (ii) prophylactic mastectomy for patients with BRCA1/2 mutations, high-risk lesions, or those who desired risk-reducing mastectomy. The exclusion criteria are severe comorbidities or inability to tolerate general anesthesia. Patients were divided into the LSPB group and the NLSPB group according to whether the SND of the affected breast was over 25 cm or the mastectomy weight was over 600 g. A detailed flowchart about patient selection is presented in Figure 1.

### R-E-NSM with DIBR

The surgical technique has been reported in our previous studies^[^25–27^]^. A 3–5 cm incision is made in the axillary skin fold through which axillary lymph node surgery, NSM, and DIBR are all accomplished. Sentinel lymph node biopsy or axillary lymph node dissection was first performed as needed under direct vision. The tissue layers dissecting will be done from deep to superficial with endoscopic techniques and air-supporting the surgical space. For prepectoral BR (Supplemental Digital Content 1, available at: http://links.lww.com/JS9/E56, Supplemental Digital Content Video 1, available at: http://links.lww.com/JS9/E56), the retromammary space is separated first, followed by the subcutaneous layer. For dual-plane BR (Supplemental Digital Content 2, available at: http://links.lww.com/JS9/E57, Supplemental Digital Content Video 2, available at: http://links.lww.com/JS9/E57), the subpectoral space is first dissected, followed by the retromammary and subcutaneous layers. Patients with malignant tumors need to excise the fascia of the pectoralis major muscle. With a minimal (just 2 mm) auxiliary incision (HUAXI Hole 1) at the superolateral margin of the areola, the inner and lower quadrants of the breast glands are entirely removed^[^29^]^. IBBR is performed immediately after R-E-NSM. The TiLOOP® Bra was placed using the “parachute method”^[^32^]^. The prosthesis was then placed under the TiLOOP® Bra via the transaxillary incision and suture on the serratus anterior.

### Air inflation adjustment technique

In the later stage, patients will undergo AIAT following BR, allowing the implant to be positioned in its standard orientation without the need for sutures or TiLoop® Braes for fixation during the procedure. The AIAT is recommended to be performed within 24 hours postoperatively and not exceeding 72 hours. The patient leans forward at 45°–90°. Air is inflated through the drainage tube, separating the implant from the muscle and skin flap, enhancing implant mobility when the implant falls with gravity and the doctor’s jiggling of the breast. Upon gas deflation, the muscle and skin layers evenly redistribute and flatten out, and the NAC resettles into its normal position, with the breasts naturally sagging as intended. Afterward, the patient wears a breast contouring garment for 3 months to promote flap attachment and uses a chest strap to fix the inframammary fold and prevent implant displacement (Supplemental Digital Content 4, available at: http://links.lww.com/JS9/E59, Supplemental Digital Content Video 3, available at: http://links.lww.com/JS9/E59).

### Follow-up

Patients were followed up in the outpatient clinic at 2 weeks, 1 month, 3 months, and every 6 months postoperatively. The data were collected until the end of the last follow-up visit on 30 September 2024. Preoperative and postoperative treatments were conducted based on breast cancer treatment guidelines. Outcome evaluation focuses on surgical safety, aesthetic outcomes, and oncologic outcomes.

Surgical safety was accessed by postoperative complications, such as hematoma, surgical site infection, incision dehiscence, skin flap/ NAC ischemia/necrosis, which were defined and graded with Clavien–Dindo classification (CDC) to be major complications (≥CDC grade III) or minor complications (CDC grade I–II)^[^32^]^.

Aesthetic outcomes include the doctor-reported outcome (Ueda scores)^[^33^]^ and the patient-reported outcome (BREAST-Q scores)^[^34^]^. For patients who have reached or exceeded 12 months after surgery, aesthetic outcomes were assessed at the 12th postoperative month, while for patients who did not, aesthetic outcome was assessed at the 6th postoperative month for the relatively stable aesthetic outcomes. The preoperatively and postoperatively photographs of reconstructive breasts were independently evaluated by three breast surgeons with no knowledge of each other’s assessments using the Ueda scale, categorizing results as “Poor,” “General,” “Good,” or “Excellent” and the final score was the average scores of three doctors. To reduce bias, an interobserver agreement test was conducted, which was found to be reliable. BREAST-Q scale was used to assess the satisfaction with breasts, psychosocial well-being, physical well-being, and sexual well-being, and the diff between the last evaluation time points and preoperative values was recorded.

Local recurrence-free survival (LRFS), distant metastasis-free survival (DMFS), and disease-free survival (DFS) were used as the primary study endpoints. LRFS was defined as the time from the surgery date to recurrence in the ipsilateral chest wall or at the breast surgical site. DMFS was defined as the time from surgery to recurrence at distant sites. DFS was defined as the time from the surgery date to recurrence, metastasis, new breast cancer on the same or opposite side, and death from any cause.

### Propensity score matching

The propensity score matching (PSM) was conducted to balance the baseline characteristics of the two groups. Matching covariates included age, laterality, axillary lymph node management, operation type, follow-up time, and treatment-related factors (neoadjuvant chemotherapy, adjuvant chemotherapy, radiotherapy), while variables directly defining breast size or ptosis (cup size, breast ptosis, SND, mastectomy weight, etc.) were excluded as they represented group-defining features (LSPB vs. NLSPB). The PSM was implemented using the R package “MatchIt”16 version 4.2.2 with 1:3 pairing, nearest neighbor methods, and a caliper of 0.1^[^25,35^]^.

### Statistical analyses

All statistical analyses were performed with R 4.2.2 and SPSS 26. Continuous variables were expressed as means with standard deviation (SD) or medians with interquartile range (IQR), while categorical variables were expressed as frequencies with percentages. The intergroup differences were compared using Student’s *t*-test, Mann–Whitney *U* test, one-way ANOVA, Chi-squared test, or Fisher exact test. Survival curves were estimated by the Kaplan–Meier method for LRFS, DMFS, and DFS, and the log-rank test was used for between-group comparisons. Two-tailed *P* values < 0.05 were considered statistically significant.

## Result

### Baseline characteristics

A total of 562 patients were initially enrolled in the study. After applying PSM to balance the baseline characteristics between the two groups, the number of participants was reduced to 344, consisting of 88 patients in the LSPB group and 256 patients in the NLSPB group (mean [SD] age: 42.6[9.4] vs. 42.7[8.3] years, *P* = 0.956) (Fig. 1). The mastectomy weight in the LSPB and NLSPB groups was 576.6 ± 144.8 g and 330.1 ± 105.7 g (*P* < 0.001). The body mass index (BMI), cup size, breast ptosis, SND, implant size, and operation time were also significantly different between the two groups (all *P* < 0.05) (Table 1).

**Table 1:** Baseline characteristics of the patients with large or severely ptotic breasts and non-large or severely ptotic breasts undergoing reverse-sequence endoscopic nipple-sparing mastectomy with direct-to-implant breast reconstruction


Characteristic	Before PSM (*n* = 562), *N* (%)			After PSM (*n* = 344), *N* (%)		
	NLSPB group (*n* = 464)	LSPB group (*n* = 98)	*P*	NLSPB group (*n* = 256)	LSPB group (*n* = 88)	*P*
Age, mean (SD), year	41.6 (9.0)	43.0 (9.6)	0.188	42.7 (8.3)	42.6 (9.4)	0.956
BMI, mean (SD), kg/m^2^	22.0 (2.6)	23.9 (3.0)	<0.001	21.9 (2.6)	24.0 (3.0)	<0.001
Smoking	5 (1.1)	1 (1.0)	0.953	4 (1.6)	1 (1.1)	0.809
Hypertension	12 (2.6)	10 (10.2)	0.001	4 (1.6)	9 (10.2)	0.001
Diabetes	6 (1.3)	4 (4.1)	0.143	2 (0.8)	4 (4.5)	0.065
Cup size			<0.001			<0.001
A	118 (25.6)	0 (0.0)		71 (28.0)	0 (0.0)	
B	234 (50.8)	0 (0.0)		127 (50.0)	0 (0.0)	
C	103 (22.3)	50 (51.0)		52 (20.5)	46 (52.3)	
D	4 (0.9)	34 (34.7)		4 (1.6)	31 (35.2)	
≥E	0 (0.0)	14 (14.3)		0 (0.0)	11 (12.5)	
Breast ptosis^a^			<0.001			<0.001
No	265 (57.9)	0 (0.0)		141 (56.2)	0 (0.0)	
I	129 (28.2)	7 (7.1)		74 (29.5)	7 (8.0)	
II	52 (11.4)	59 (60.2)		27 (10.8)	55 (62.5)	
III	7 (1.5)	30 (30.6)		4 (1.6)	24 (27.3)	
The distance from the sternal notch to the nipple, mean (SD), cm	19.4 (2.7)	29.3 (2.2)	<0.001	19.3 (2.7)	29.2 (2.2)	<0.001
Laterality			0.681			0.604
Unilateral	390 (84.1)	84 (85.7)		221 (86.3)	74 (84.1)	
Bilateral	74 (15.9)	14 (14.3)		35 (13.7)	14 (15.9)	
Prophylactic resection^b^			0.617			0.593
Yes	17 (3.7)	2 (2.0)		11 (4.3)	2 (2.3)	
No	447 (96.3)	96 (98.0)		245 (95.7)	86 (97.7)	
Mastectomy weight, mean (SD), g	332.0 (103.4)	573.5 (143.4)	<0.001	330.1 (105.7)	576.6 (144.8)	<0.001
Implant size, mean (SD), g	286.5 (76.2)	424.4 (78.0)	<0.001	286.2 (79.7)	425.3 (77.8)	<0.001
Operation time, median (IQR), minutes	146.0 (117.8, 188.2)	157.0 (128.0, 206.5)	0.062	146.5 (118.0, 182.0)	160.0 (129.0, 211.2)	0.031
Unilateral	138.0 (113.0, 180.0)	150.5 (120.0, 201.5)	0.071	142.0 (114.0, 180.0)	152.0 (123.8, 203.8)	0.067
Bilateral	177.5 (150.2, 223.0)	205.5 (160.2, 256.2)	0.209	175.0 (147.5, 217.0)	205.5 (160.2, 256.2)	0.219
Operation type			0.721			0.682
PBR	256 (55.2)	56 (57.1)		139 (54.3)	50 (56.8)	
Dual-plane BR	208 (44.8)	42 (42.9)		117 (45.7)	38 (43.2)	
Axillary lymph node management			0.110			0.954
Untreated	11 (2.4)	3 (3.1)		6 (2.3)	2 (2.3)	
SLNB	320 (69.0)	58 (59.2)		171 (66.8)	57 (64.8)	
ALND ± SLNB	133 (28.7)	37 (37.8)		79 (30.9)	29 (33.0)	
Nipple resection	24 (5.2)	6 (6.1)	0.704	17 (6.6)	6 (6.8)	0.954
AIAT			0.373			0.860
Yes	305 (65.7)	69 (70.4)		169 (66.0)	59 (67.0)	
No	159 (34.3)	29 (29.6)		87 (34.0)	29 (33.0)	
Hospitalization			0.949			0.855
Day surgery	273 (58.8)	58 (59.2)		157 (61.3)	53 (60.2)	
Inpatient surgery	191 (41.2)	40 (40.8)		99 (38.7)	35 (39.8)	
Hospital stays, median (IQR), days	1.0 (1.0, 6.0)	1.0 (1.0, 5.0)	0.746	1.0 (1.0, 6.0)	1.0 (1.0, 5.0)	0.913
Hospital cost, mean (SD), CNY	55850.4 (15663.3)	54173.5 (14907.3)	0.332	55589.9 (15143.3)	54990.7 (15493.3)	0.751
Pathologic T stage (NA = 5)^c^,^d^			0.015			0.168
Tis	64 (13.9)	11 (11.2)		33 (13.0)	11 (12.5)	
T1	185 (40.3)	31 (31.6)		100 (39.5)	30 (34.1)	
T2	184 (40.1)	54 (55.1)		105 (41.5)	45 (51.1)	
T3	4 (0.9)	0 (0.0)		2 (0.8)	0 (0.0)	
Pathologic N stage (NA = 2)^c^,^e^			0.053			0.678
N0	345 (74.7)	68 (69.4)		180 (70.6)	64 (72.7)	
N1	88 (19.0)	23 (23.5)		57 (22.4)	19 (21.6)	
N2	5 (1.1)	3 (3.1)		4 (1.6)	2 (2.3)	
N3	4 (0.9)	2 (2.0)		2 (0.8)	1 (1.1)	
ER (NA = 20)^c^			0.311			0.237
Positive	325 (72.4)	65 (69.9)		188 (74.9)	61 (71.8)	
Negative	107 (23.8)	26 (28.0)		52 (20.7)	22 (25.9)	
PR (NA = 35)^c^			0.385			0.347
Positive	292 (67.1)	60 (65.2)		172 (69.9)	57 (67.9)	
Negative	126 (29.0)	30 (32.6)		63 (25.6)	25 (29.8)	
HER2 (NA = 36)^c^			0.420			0.526
Positive^f^	102 (23.5)	28 (30.4)		56 (22.9)	25 (29.4)	
Negative	314 (72.4)	62 (67.4)		178 (72.7)	58 (68.2)	
Ki-67 (NA = 36)			0.681			0.916
≤20	204 (47.0)	48 (52.2)		118 (48.4)	44 (52.4)	
>20	213 (49.1)	42 (45.7)		115 (47.1)	38 (45.2)	
Neoadjuvant chemotherapy	79 (17.0)	29 (29.6)	0.004	51 (19.9)	21 (23.9)	0.433
Adjuvant chemotherapy (NA = 37)	229 (53.5)	48 (49.5)	0.474	131 (51.2)	45 (51.1)	0.995
Radiotherapy (NA = 23)	120 (27.1)	27 (28.1)	0.836	64 (25.0)	24 (27.3)	0.673
Adjuvant endocrino therapy (NA = 1)	348 (75.2)	75 (76.5)	0.775	198 (77.3)	70 (79.5)	0.668
Anti-HER2 therapy (NA = 56)	107 (26.1)	26 (27.1)	0.843	64 (26.1)	23 (26.1)	0.998
Follow-up time, median (IQR), months	21.9 (9.0, 33.0)	19.7 (10.1, 30.0)	0.899	23.0 (10.6, 32.2)	21.0 (13.8, 32.4)	0.889
Ueda score evaluation time points^g^			0.883			0.116
6th months	63 (19.0)	16 (19.8)		38 (19.7)	12 (16.0)	
12th months	268 (81.0)	65 (80.4)		155 (80.3)	63 (84.0)	
Breast-Q score evaluation time points^h^			0.822			0.552
6th months	64 (20.4)	16 (21.6)		40 (22.0)	13 (18.6)	
12th months	249 (79.6)	58 (78.4)		142 (78.0)	57 (81.4)	


AIAT, air inflation adjustment technique; ALND, axillary lymph node dissection; BMI, body mass index; Dual-plane BR, subpectoral dual-plane implant-based breast reconstruction; ER, estrogen receptor; HER-2, human epidermal growth factor receptor 2; IQR, interquartile range; LSPB, large or severely ptotic breasts; NA, not available; NLSPB, non-large or severely ptotic breasts; PBR, prepectoral implant-based breast reconstruction; PR, progesterone receptor; PSM, propensity score matching; SD, standard deviation; SLNB, sentinel lymph node biopsy.

^a^ 7 cases of pseudosagosis before PSM and after PSM.

^b^ Only bilateral prophylactic mastectomy is considered prophylactic mastectomy.

^c^ only including malignant cases (543 cases were malignant before PSM and 331 cases were malignant after PSM).

^d^ 5 cases of Tx stage before PSM and 2 cases of Tx stage after PSM.

^e^ 1 case of Nx stage, 1 case of sn stage, 1 case of 1 min stage before PSM, and 1 case of 1 min stage after PSM.

^f^ including carcinoma in situ.

^g^ Aesthetic assessments captured the difference between the 6th and 12th month and preoperative values. Before PSM, Ueda score NA = 150, after PSM, Ueda score NA = 76.

^h^ Aesthetic assessments captured the difference between the 6th and 12th month and preoperative values. Before PSM, Breast-Q score in satisfaction with breast NA = 175, Breast-Q score in psychological well-being NA = 181, Breast-Q score in physical well-being NA = 188, Breast-Q score in sexual well-being NA = 128. After PSM, Breast-Q score in satisfaction with breast NA = 92, Breast-Q score in psychological well-being NA = 97, Breast-Q score in physical well-being NA = 97, Breast-Q score in sexual well-being NA = 100.

### Surgical safety

Before PSM, no significant differences were observed in surgical complications between the two groups, except that the rate of flap necrosis (3.1% vs. 0.2%, *P* = 0.009), NAC ischemia (4.1% vs. 0.4%, *P* = 0.001) and CDC ≥ II (24.5% vs. 13.0%, *P* = 0.004) in the LSPB group was significantly higher than in the NLSPB group. After PSM, apart from the rate of NAC ischemia in the LSPB group was higher than in the NLSPB group (4.5% vs. 0.8%, *P* = 0.020), there were no significant differences in any complications, major complications, minor complications between the two groups (all *P* > 0.05) (Table 2).

**Table 2:** The surgical complications and implant-related complications of the patients with large or severely ptotic breasts and non-large or severely ptotic breasts undergoing reverse-sequence endoscopic nipple-sparing mastectomy with direct-to-implant breast reconstruction


Variables	Before PSM (*n* = 562), *N* (%)			After PSM (*n* = 344), *N* (%)		
	NLSPB group (*n* = 464)	LSPB group (*n* = 98)	*P*	NLSPB group (*n* = 256)	LSPB group (*n* = 88)	*P*
Surgical complications						
Any surgical complications	89 (19.2)	27 (27.6)	0.063	58 (22.7)	24 (27.3)	0.381
Major complications (CDC ≥ IIIa)	13 (2.8)	6 (6.1)	0.179	8 (3.1)	6 (6.8)	0.230
Implant loss	8 (1.7)	2 (2.0)	1.000	6 (2.3)	2 (2.3)	1.000
Flap necrosis (reoperation)	4 (0.9)	5 (5.1)^a^	0.009	4 (1.6)	5 (5.7)^a^	0.089
NAC ischemia (reoperation)	2 (0.4)	4 (4.1)	0.001	2 (0.8)	4 (4.5)	0.020
Wound dehiscence (reoperation)	6 (1.2)	0 (0.0)	0.555	2 (0.8)	0 (0.0)	1.000
SSI (reoperation)	5 (1.1)	2 (2.0)	0.779	3 (1.2)	2 (2.3)	0.820
CDC ≥ IIIb	8 (1.7)	3 (3.1)	0.641	6 (2.3)	3 (3.4)	0.878
Minor complications (CDC I-II)	81 (17.5)	21 (21.4)	0.354	54 (21.1)	18 (20.5)	0.889
Hematoma/Hemorrhage	14 (3.0)	3 (3.1)	1.000	10 (3.9)	3 (3.4)	1.000
SSI	57 (12.3)	17 (17.3)	0.178	37 (14.5)	14 (15.9)	0.740
Wound dehiscence	5 (1.1)	1 (1.0)	1.000	5 (2.0)	1 (1.1)	0.974
Flap ischemia	6 (1.3)	2 (2.0)^b^	0.922	1 (0.4)	1 (1.1)^b^	0.447
NAC ischemia	7 (1.5)	1 (1.0)	0.711	5 (2.0)	1 (1.1)	0.614
CDC ≥ II	60 (12.9)	24 (24.5)	0.004	40 (15.6)	21 (23.9)	0.081
Repeated SSI	12 (2.6)	5 (5.1)	0.319	6 (2.3)	4 (4.5)	0.488
Readmitted	13 (2.8)	8 (8.2)	0.024	9 (3.5)	7 (8.0)	0.158
Implant-related complications						
Implant-related complications	108 (23.3)	20 (20.4)	0.539	64 (25.0)	19 (21.6)	0.519
Rippling (NA = 12)^c^	31 (6.9)	2 (2.0)	0.069	18 (7.2)	2 (2.3)	0.091
Prosthesis outline appearance (NA=8)			0.931			0.962
Mild	25 (5.5)	6 (6.1)		13 (5.2)	6 (6.8)	
Moderate	18 (3.9)	1 (1.0)		12 (4.8)	1 (1.1)	
Severe	5 (1.1)	3 (3.1)		3 (1.2)	3 (3.4)	
Capsular contraction (NA = 7)			0.286			0.362
Baker III-IV	24 (5.2)	8 (8.2)		15 (5.9)	8 (9.1)	
Prosthesis rotation (NA = 10)	10 (2.2)	1 (1.0)	0.718	8 (3.2)	1 (1.1)	0.516


CDC, Clavien–Dindo classification; LSPB, large or severely ptotic breasts; NA, not available; NAC the nipple-areola complex; NLSPB, non-large or severely ptotic breasts; PSM, propensity score matching; SSI, surgical site infection.

^a^ In major compilations, one patient experienced flap necrosis due to improper postoperative compression care, but she recovered after re-suturing.

^b^ In minor compilations, one patient experienced flap ischemia due to improper postoperative compression care, but she recovered after re-suturing.

^c^ The rippling observed in the standing position is considered the standard, and the rippling observed in the forward bending position is not included.

### Aesthetic outcomes

No matter before or after PSM, the implant-related complication rate in the LSPB group was consistently not significantly different from that in the NLSPB group (before PSM: 20.4% vs. 23.3%, *P* = 0.566; after PSM: 21.6% vs. 25.0%, *P* = 0.519) (Table 2).

After PSM, the difference in the evaluation time points distribution of the two groups was not significant for Ueda scores (*P* = 0.116) and BREAST-Q scores (*P* = 0.552) (Table 1). Ueda scores in the NLSPB group were significantly superior to those in the LSPB group (*P* = 0.026), but no significant differences were observed in BREAST-Q score changes between the two groups (all *P* > 0.050) (Table 3).

**Table 3:** Aesthetic outcomes of the patients with large or severely ptotic breasts and non-large or severely ptotic breasts undergoing reverse-sequence endoscopic nipple-sparing mastectomy with direct-to-implant breast reconstruction

Variables	Before PSM (*n* = 562), *N* (%)			After PSM (*n* = 344), *N* (%)		
	NLSPB group (*n* = 464)	LSPB group (*n* = 98)	*P*	NLSPB group (*n* = 256)	LSPB group (*n* = 88)	*P*
Ueda Score^a^						
Excellent	234 (70.7)	41 (50.6)	0.001	129 (66.8)	39 (52.0)	0.026
Good	78 (23.6)	33 (40.7)		52 (26.9)	29 (38.7)	
General	15 (4.5)	5 (6.2)		9 (4.7)	5 (6.7)	
Poor	4 (1.2)	2 (2.5)		3 (1.6)	2 (2.7)	
Breast-Q Score^a^						
Satisfaction with breast	0.0 (−12.0, 11.0)	6.0 (−6.0, 15.0)	0.025	1.0 (−11.8, 14.0)	6.0 (−5.5, 15.0)	0.087
Psychological well-being	0.0 (−16.0, 16.2)	0.0 (−11.0, 10.0)	0.713	0.0 (−16.0, 16.0)	0.0 (−10.0, 10.0)	0.692
Physical well-being	−8.0 (−26.0, 10.0)	−11.0 (−25.2, 8.5)	0.892	−4.0 (−26.0, 12.0)	−9.5 (−22.0, 8.5)	0.515
\Sexual well-being^b^	−7.0 (−26.0, 13.0)	−7.0 (−17.0, 5.0)	0.751	−7.0 (−26.0, 13.5)	−7.0 (−17.0, 6.0)	0.776


IQR, interquartile range; LSPB, large or severely ptotic breasts; NLSPB, non-large or severely ptotic breasts; PSM, propensity score matching

^a^ Aesthetic assessments captured the difference between the 6th and 12th month and preoperative values. Before PSM, Ueda score NA = 150, after PSM, Ueda score NA = 76. Before PSM, Breast-Q score in satisfaction with breast NA = 175, Breast-Q score in psychological well-being NA = 181, Breast-Q score in physical well-being NA = 188, Breast-Q score in sexual well-being NA = 128. After PSM, Breast-Q score in satisfaction with breast NA = 92, Breast-Q score in psychological well-being NA = 97, Breast-Q score in physical well-being NA = 97, Breast-Q score in sexual well-being NA = 100.

^b^ 7 cases were not sexually active before PSM and 4 cases were not sexually active after PSM.

**Figure 2.**
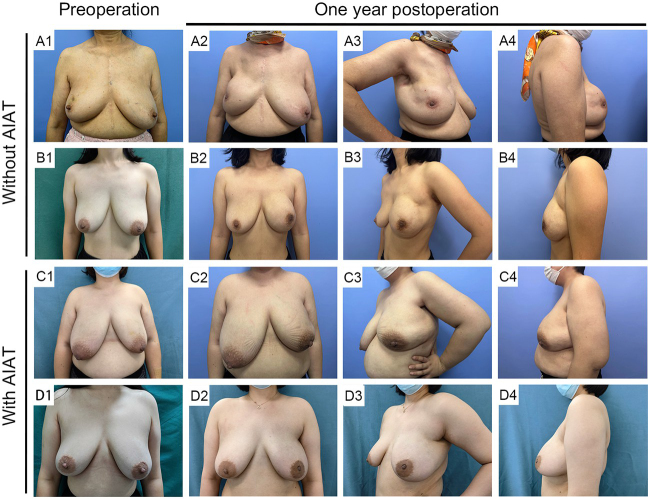
The preoperative and postoperative photos of the patients with large or severely ptotic breasts undergoing reverse-sequence endoscopic nipple-sparing mastectomy with direct-to-implant breast reconstruction with and without air inflation adjustment technique. (A1, B1) The preoperative photos of the patients without AIAT. (A2–A4, B2–B4) The postoperative photos of the patients without AIAT. (C1, D1) The preoperative photos of the patients with AIAT. (C2–C4, D2–D4) The postoperative photos of the patients with AIAT. AIAT, air inflation adjustment technique.

Upon further examination, it was observed that within the LSPB group, there was no statistically significant variation in Ueda scores between patients who underwent AIAT and those who did not (*P* = 0.110). Nevertheless, the proportion of patients achieving top-tier ratings of “excellent” or “good” was notably higher among those who received AIAT, with a comparison of 93.7%–85.1%. Moreover, no significant difference was found in Ueda scores when comparing the LSPB group treated with AIAT to the NLSPB group without AIAT (*P* = 0.870). Interestingly, the LSPB group with AIAT exhibited a markedly higher increase in BREAST-Q scores for breast satisfaction compared to the NLSPB group without AIAT, with statistical significance (P = 0.004) (Table 4). Preoperative and postoperative photographs are displayed in Figure 2.

**Table 4:** Comparison of aesthetic outcomes with and without air inflation adjustment technique for patients with large or severely ptotic breasts and non-large or severely ptotic breasts undergoing reverse-sequence endoscopic nipple-sparing mastectomy with direct-to-implant breast reconstruction

Variables	Without AIAT (*n* = 116), *N* (%)			With AIAT (*n* = 228), *N* (%)				
	NLSPB group (*n* = 87)	LSPB group (*n* = 29)	*P*_1_^a^	NLSPB group (*n* = 169)	LSPB group (*n* = 59)	*P*_2_^a^	*P*_3_^a^	*P*_4_^a^
Ueda Score^b^								
Excellent	44 (57.9)	11 (40.7)	0.120	85 (72.6)	28 (58.3)	0.078	0.110	0.870
Good	25 (32.9)	12 (44.4)		27 (23.1)	17 (35.4)			
General	6 (7.9)	3 (11.1)		3 (2.6)	2 (4.2)			
Poor	1 (1.3)	1 (3.7)		2 (1.7)	1 (2.1)			
Breast-Q Score^b^								
Satisfaction with breast	−2.0 (−24.0, 10.5)	3.5 (−8.0, 16.5)	0.073	3.0 (−5.2, 14.5)	6.0 (−2.2, 14.8)	0.363	0.421	0.004
Psychological well-being	0.0 (−21.2, 16.0)	0.0 (−9.0, 30.0)	0.150	0.0 (−12.2, 15.2)	0.0 (−11.5, 6.8)	0.496	0.479	0.531
Physical well-being	−8.0 (−32.0, 15.0)	0.0 (−30.0, 15.0)	0.777	0.0 (−21.0, 11.5)	−12.0 (−18.0, 5.0)	0.222	0.343	0.728
Sexual well-being^c^	−8.5 (−27.5, 6.5)	−5.0 (−13.5, 8.0)	0.409	−5.0 (−26.0, 16.0)	−8.0 (−19.5, 0.0)	0.844	0.445	0.750


AIAT, air inflation adjustment technique; LSPB, large or severely ptotic breasts; NLSPB, non-large or severely ptotic breasts.

^a^ P_1_ represents the comparison between the LSPB and NLSPB groups without AIAT, P_2_ represents the comparison between the LSPB and NLSPB groups with AIAT, P_3_ represents the comparison of the LSPB group without and with AIAT, P4 represents the comparison between the NLSPB group without AIAT and LSPB group with AIAT.

^b^ Aesthetic assessments captured the differences between the last follow-up and preoperative values. Before AIAT, Ueda score NA = 13, Breast-Q score in satisfaction with breast NA = 22, Breast-Q score in psychological well-being NA = 25, Breast-Q score in physical well-being NA = 24, Breast-Q score in sexual well-being NA = 27. After AIAT, Ueda score NA = 63, Breast-Q score in satisfaction with breast NA = 70, Breast-Q score in psychological well-being NA = 72, Breast-Q score in physical well-being NA = 73, Breast-Q score in sexual well-being NA = 73.

^c^ 3 cases were not sexually active.

### Oncologic outcomes

**Figure 3.**
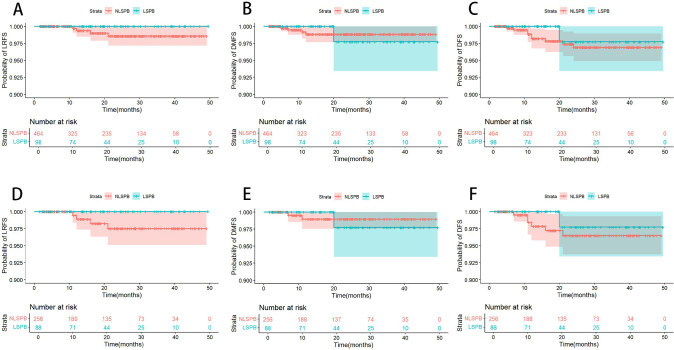
Kaplan–Meier survival curve of oncologic outcomes in the LSPB and NLSPB groups before and after propensity score matching. (A) The probability of LRFS between two groups before PSM. (B) The probability of DMFS between two groups before PSM. (C) The probability of DFS between two groups before PSM. (D) The probability of LRFS between two groups after PSM. (E) The probability of DMFS between two groups after PSM. (F) The probability of DFS between two groups after PSM. The red arrow indicates the indentation caused by pressure. DFS, disease-free survival; DMFS, distant metastasis-free survival; LRFS, local recurrence-free survival; LSPB, large or severely ptotic breasts; NLSPB, non-large or severely ptotic breasts; PSM, propensity score matching.

The median (IQR) follow-up time was 21.9 (9.0, 33.0) and 19.7 (10.1, 30.0) months in the LSPB and NLSPB groups. Before PSM, the LSPB group had no local recurrences and one case of distant metastasis, while the NLSPB group had four cases of local recurrences and four cases of distant metastases. No matter before or after PSM, the *P* values of LRFS, DMFS, and DFS were not significantly different between the two groups (Fig. 3).

## Discussion

Contrary to the previous view that LSPB was not recommended or even relatively contraindicated for open NSM, conventional E-NSM, and R-NSM, our study surprisingly discovered that the R-E-NSM with DIBR and AIAT can bring favorable surgical safety and aesthetic outcomes in patients with LSPB, similar or even superior to NLSPB patients without AIAT. This breakthrough overcomes the indication limitations of traditional E-NSM, R-NSM, and even open NSM with DIBR. Thus, we propose that LSPB should not be a contraindication for NSM with DIBR. R-E-NSM with DIBR offers a new surgical option for patients with LSPB beyond the NSM with WST, especially for those who do not need breast ptosis correction.

As expected, compared to the NLSPB group, patients in the LSPB group had significantly higher BMI, cup size, breast ptosis, mastectomy weight, and implant size^[^3,10^]^. More hypertensive patients and relatively more diabetes patients in the LSPB group may be associated with higher BMI^[^36,37^]^. The large area of skin flap and difficulty of gland excision may account for the longer operative time in the LSPB group. The operative time in the LSPB group in the study was comparable to the average operative time previously reported for NSM with WST and immediate prepectoral BR with implant or tissue expander in LSPB, with a slightly shorter operative time for unilateral surgery in this study (155 vs. 152 minutes)^[^38^]^. The mastectomy weight in the LSPB group was 576.6 ± 144.8 g in our study, exceeding the results reported in several studies about NSM in patients with LSPB, as well as the 557.11 g defined for large breasts^[^11,38,39^]^.

**Figure 4.**
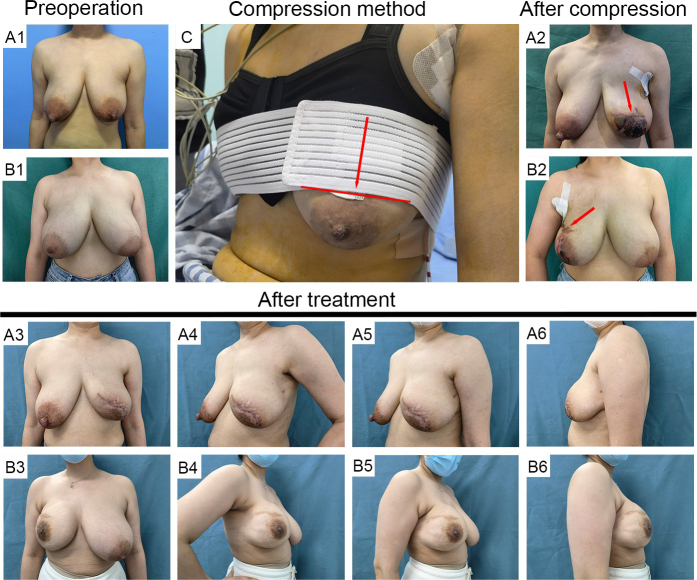
Patients experienced flap necrosis due to incorrect pressure care management. (A1, B1) The preoperative photos of patients. (A2, B2) The postoperative photos of patients with flap necrosis due to incorrect pressure care management. (A3-A6, B3-B6) The postoperative photos of improvement after treatment. (C) Postoperative compression method.

Surgical safety is an essential consideration for NSM in LSPB patients. Frey *et al*^[^3^]^ found that reconstructive and ischemic complications significantly increased with the resected gland weight and breast size in open NSM. A systematic review including 31 studies about BR of LSPB reported a 29.08% overall complication rate, with an average mastectomy weight of 557.11 g^[^11^]^. In our study, patients with LSPB also had a higher rate of NAC ischemia (reoperation) than NLSPB, but both rates were relatively low. We think the higher rate may be related to the inherent and intrinsic disadvantages present in LSPB patients, as previously discussed, which could be minimized by technical advances, but will not completely disappear. Besides, two ischemia or necrosis cases were attributed to excessive postoperative compression, which was used to shape breast ptosis before the AIAT was invented, and both of them recovered with timely treatment (Fig. 4). Excluding these cases, the difference in the NAC ischemia (reoperation) rate was no longer significant (LSPB 3.5% vs. NLSPB 0.8%, *P* = 0.197). Additionally, the two groups have no significant difference in the rate of any and major complications. The complication rate in LSPB patients undergoing R-E-NSM with DTI-BR is significantly lower than that in patients with small breasts who have undergone conventional open NSM with DTI-BR. Moreover, it is either lower than or comparable to the complication rate seen in those with small breasts undergoing minimal access NSM with DTI-BR^[^20,40,41^]^. The incidence of nipple ischemia, flap ischemia/necrosis, and incision dehiscence were lower than those of NSM with WST^[^11,38^]^. These outcomes stem from R-E-NSM’s advanced technology with DIBR, notably the reverse-sequence dissection and air-insufflation methods using CO_2_, which stabilized the surgical environment. This precision allowed for meticulous dissection of the breast tissue along the superficial layer of the superficial fascia, ensuring uniform flap thickness and protecting the blood supply to the skin flap and NAC^[^4,42–44^].^ As a result, the procedure maintained oncological safety while enhancing surgical accuracy. The incision location is another significant risk factor for ischemic complications^[^14,45^]^. However, the R-E-NSM technique takes an axillary approach, with no incision on the breast surface, which virtually avoids skin trophoblastic vascular dissociation and incisional dehiscence. Besides, the implant’s gravity may also pull the breast and compress the skin flap^[^46^].^ However, during the R-E-NSM with DTI-BR procedure, the TiLoop® Bra can be easily placed with our “parachute” method, enabling the breast to tolerate the heavy implant^[^26^]^. At last, the increased surgical efficiency also helps to reduce the infection risk^[^47^]^.

Regarding aesthetic outcomes, there was no significant difference in implant-related complications between the two groups. For Ueda scores, we found that the absolute values of excellent and good rates were higher in the LSPB group with AIAT than in both LSPB and NLSPB groups without AIAT, although there was no significant difference. However, the LSPB group with AIAT had a significantly higher increase in BREAST-Q scores of breast satisfaction than the NLSPB group without AIAT. These findings may indicate that AIAT helps improve breast satisfaction for LSPB patients. In R-E-NSM for LSPB patients, though breasts become scarless, the long skin envelope and the around friction prevent the implant from being pushed into the proper position^[^48^]^. With AIAT, the skin and muscle can redistribute evenly on the implant’s surface instead of stacking, and the implant and nipple position can be easily adjusted to the optimal state, improving the reconstructed breasts’ aesthetics and the bilateral breasts’ symmetry. We believe that uniform intraoperative breast flap dissection and flattening of the muscle and skin on the implant surface in the short postoperative period via AIAT may be potentially beneficial for the reduction of the rippling in the long-term postoperative period. Furthermore, in contrast to previous concerns about seroma in the operative area, patients are encouraged to move their upper limbs postoperatively to promote the production of fluid in the skin envelope, which helps to create a loose peri-implant capsule, making the reconstructed breast soft to the touch, and may also reduce the incidence of capsular contraction and rippling. The Ueda scores focus on the appearance symmetry of bilateral breasts’ position, color, and shape, while the BREAST-Q score focuses on the size, position, and softness of the reconstructed breast as well as the appearance of bilateral breasts with clothes or bra on^[^33,34,49^]^. Thus, the difference in BREAST-Q between the LSPB group with AIAT and the NLSPB groups without AIAT is more prominent. The postoperative breast appearance of R-E-NSM is different from NSM with WST in that the breasts are still ptotic^[^50,51^]^. Therefore, for those needing breast ptosis correction, the NSM with WST may be the best option, while our approach may be superior for those who don’t.

The data captures a tendency for lower local recurrence in LSPB patients. This may be because large breasts have more glands, and tumors are less likely to grow beyond the glands’ surface and the retromammary space. However, the same size tumor may be unbearable for a small breast, which is prone to skin and chest wall invasion. Further follow-up is still required for long-term assessment of oncologic safety.

There are some strengths in our study. First, we are the globally first article to explore NSM in patients with LSPB with endoscopic techniques. Second, this study is a prospective cohort and has a substantial amount of data about minimal invasive mastectomy. Third, PSM analysis was used to correct for differences in baseline characteristics. Fourth, the R-E-NSM with DIBR procedure we adopted is an original minimally invasive surgery with the technical advantages of being efficient, scarless, and safe. The technique breaks through the limitations of BR layers in the conventional E-NSM and R-NSM and fully realizes and even surpasses those in open NSM, such as prepectoral and dual plane BR for LSPB, except for procedures that require skin excision. We also have some limitations. It is a single-center study. There is a learning curve for endoscopic techniques. The existing favorable performance of R-E-NSM is inextricably linked to clinical practice and refinement by our team, which it had to cross the learning curve to achieve. A longer follow-up is needed for surgical and aesthetic complications and oncologic outcomes.

## Conclusion

R-E-NSM with DIBR can bring favorable and the same safety and aesthetic outcomes for patients with LSPB compared with NLSPB patients. The AIAT helps to improve breast aesthetics for patients with LSPB. R-E-NSM with DIBR offers a new surgical option for patients with LSPB beyond the NSM with WST, especially for those who do not require breast ptosis correction.

## Data Availability

Datasets generated during and/or analyzed during the current study are not publicly available, but available upon reasonable request.

## Research registration unique identifying number (UIN)

Name of the registry: Chinese Clinical Trial Registry. Unique Identifying number or registration ID: ChiCTR2100047081. Hyperlink to your specific registration (must be publicly accessible and will be checked): https://www.chictr.org.cn/showproj.html?proj=127254.

## Presentation

This study was previously presented as a poster presentation at 2024 San Antonio Breast Cancer Symposium. Details are as follows: P4-09-07: Clinical Outcomes of Reverse‑Sequence Endoscopic Nipple‑Sparing Mastectomy with Direct‑to‑Implant Breast Reconstruction in Breast Cancer Patients with Large or Severe Ptotic Breast: A Single-center Prospective Cohort Study (Abstract Number: SESS-3678).
